# Deltoid Ligament Augmentation Replacing Syndesmotic Fixation for the Treatment of Ankle Fracture With Syndesmotic Instability and Deltoid Ligament Rupture: A Cadaveric Study

**DOI:** 10.1111/os.70219

**Published:** 2025-12-12

**Authors:** Fei Han, Liu Yan, Li Ting, Wang Jun, Sun Zhijian, Li Changrun, Zhang Weiguang, Liu Huaicun, Ding Huiru, Huan Yong

**Affiliations:** ^1^ Department of Orthopedic Trauma, Beijing Jishuitan Hospital Capital Medical University Beijing China; ^2^ State Key Laboratory of Nonlinear Mechanics, Chinese Academy of Science Beijing China; ^3^ Shenzhen Second People's Hospital The First Affiliated Hospital of Shenzhen University Shenzhen China; ^4^ Department of Anatomy Peking University Beijing China

**Keywords:** ankle fracture, biomechanics, deltoid ligament, syndesmosis, trauma

## Abstract

**Objectives:**

Ankle fracture with both deltoid ligament (DL) rupture and syndesmotic diastasis is often treated by syndesmotic fixation after fibular fixation. However, a second operation may be needed to remove the internal fixation, and screw breakage/misplacement may occur. The present study aimed to explore the mechanism and feasibility of DL augmentation instead of syndesmotic fixation from the perspective of biomechanics.

**Methods:**

The CT data (in DICOM format) of a 33‐year‐old man were used to create a finite element model. External rotation stress and eversion stress were applied to the model, and the medial clear space (MCS) and tibiofibular clear space (TCS) were evaluated. In a separate experiment, preserved lower limb specimens were fixed on a hydraulic loading frame before undergoing DL augmentation and syndesmotic fixation in random order. A mechanical testing device was used to apply external rotation stress (4 N·m) and eversion stress (2.5 N·m) to the two groups (DL augmentation or syndesmotic fixation). The MCS and TCS were measured and compared between the two groups.

**Results:**

In the finite element study, the MCS widening was lesser and the TCS widening was greater in the DL augmentation group than in the syndesmotic fixation group in both the external rotation and eversion tests. Nine specimens were analyzed in the biomechanical tests. There were no significant differences between the two groups in the widening of the TCS in the rotation tests (*p* = 0.093, Hodges–Lehmann median difference = −0.79, 95% confident interval: −1.70~0.27) and eversion tests (*p* = 0.237, HLD = −0.84, 95% CI: −2.57~1.09). However, the widening of the MCS was significantly lesser in the DL augmentation group than in the syndesmotic fixation group during the rotation tests (*p* = 0.036, HLD = 3.57, 95% CI: 0.40~6.41) and eversion tests (*p* = 0.018, HLD = 4.36, 95% CI: 1.84~7.35).

**Conclusions:**

Compared with syndesmotic fixation, DL augmentation has better resistance to medial malleolar space widening under both external rotation and eversion forces and can restore the tibiofibular space to a certain extent. These results suggest that DL augmentation alone is a potential alternative to syndesmotic fixation for Weber‐type C ankle fractures from a biomechanical point of view.

## Graphical abstract

A biomechanical study verifying that deltoid ligament augmentation can be an alternative to syndesmotic fixation for stabilizing both the medial malleolar and the syndesmosis in selective patients with Weber C ankle injuries.

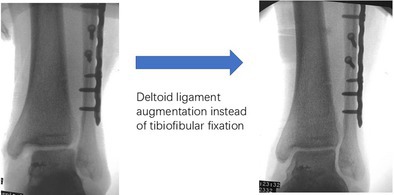

## 1 Introduction

Ankle fracture with syndesmotic injury and deltoid ligament (DL) rupture is a highly unstable injury. Traditionally, the syndesmosis could be stabilized with distal syndesmotic fixation [1, 2]. Furthermore, evidence suggests that DL repair is unnecessary after fibular reduction and syndesmotic fixation, and that the DL heals indirectly with good clinical results [3, 4, 5, 6, 7]. However, distal syndesmotic fixation has several disadvantages. First, although most surgeons now do not routinely remove the tibiofibular screws, these screws could fail and break after long‐term loading; hence, some surgeons prefer to remove the syndesmotic screws in clinical practice in some countries [8, 9]. However, a second operation consumes medical resources and costs and increases the surgical risks. Additionally, screw breakage caused by early weight‐bearing often causes failure of screw removal and may lead to medical disputes. Second, the patient cannot weight‐bear for at least 6 weeks after syndesmotic fixation [8, 9], which is not conducive to functional exercise and rehabilitation, and gives rise to joint stiffness, muscle atrophy, and osteoporosis. Third, malreduction and screw breakage can occur during the placing of the tibiofibular screws [10, 11, 12, 13].

Previous studies have reported alternative methods of traditional tibiofibular screws, e.g., absorbable tibiofibular screws, to avoid the need for screw removal [14, 15, 16]. However, absorbable screw placement still carries risks of malreduction and screw misplacement, and patients still cannot weight‐bear in the early postoperative period, which negatively affects rehabilitation. Additionally, there may be risks of tibiofibular fusion and foreign body reaction [16]. The tibiofibular joint can also be stabilized using endo‐buttons [17, 18, 19], which allows patients to resume activity soon after surgery without the need for routine removal. However, endo‐buttons have potential issues with malreduction, skin irritation, osteolysis, submergence, and ectopic ossification [20, 21] and do not resolve the problems associated with the lack of early weight‐bearing and functional recovery [22].

Clinical experience and research have demonstrated that fibular fixation with simple DL repair for Danis‐Weber‐type C ankle fractures results in a similar outcome to syndesmotic fixation. Additionally, some studies report that DL repair without syndesmotic fixation achieves satisfactory clinical results [23, 24, 25, 26]. However, the exact mechanism of this phenomenon is unclarified.

Based on the literature and our clinical findings, we hypothesized that DL augmentation alone for Weber‐type C ankle injuries biomechanically stabilizes the medial column injury without the need for syndesmotic fixation. Since 2014, we have treated more than 50 patients with such ankle fractures using DL augmentation instead of syndesmotic fixation and achieved satisfactory clinical outcomes at an average follow‐up of 24 ± 12 months (range 6–40 months) [27]. In the present study, we aimed to compare the external rotation and eversion stability after DL augmentation versus syndesmotic fixation in a cadaveric ankle fracture model to reveal the biomechanical mechanism underlying this phenomenon.

## 2 Materials and Methods

### 2.1 Model Establishment and Grouping Method

In the syndesmotic fixation group, the syndesmosis was reduced using a clamp with the ankle joint in a neutral position. The screw was inserted in a standard manner through a pre‐drilled hole created 2 cm proximal to the tibiofibular joint. A 3.5‐mm cortex screw was inserted with tricortical engagement, as used in clinical practice.

In the DL augmentation group, a 2.5‐mm Kirschner guidewire (K‐wire) was used to drill a hole at the center of the deep DL insertion at the talus, parallel to the talus dome, followed by the placement of a 5.0‐mm suture anchor (Tianxing, Beijing, China). Two oblique holes (one at the inter‐colliculus groove level and one at the posterior colliculus) were drilled through the medial malleolus using a 2.0‐mm K‐wire in the direction of the DL insertion. The four wires connected to the anchor were then drawn out through the two holes, with two sutures in each hole. Finally, the suture was secured on the medial tibial cortex at the ankle in an inversion position, while the superficial DL was sutured directly if possible.

### 2.2 Finite Element Analysis

**FIGURE 1:**
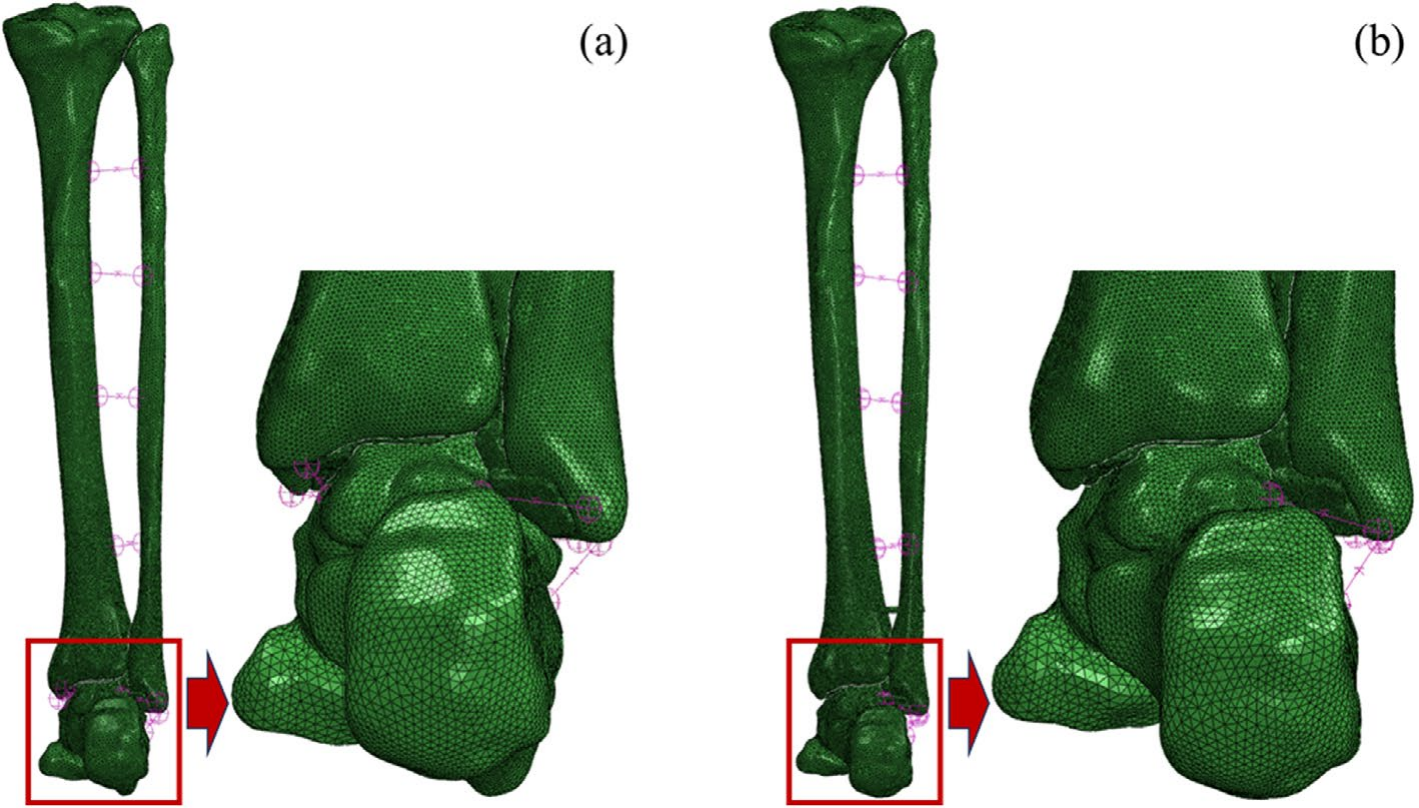
Finite element models of the ankle joint. (a) Ligament method, (b) Screw method.

Before the biomechanical tests, a finite element study was conducted using the CT data (in DICOM format) of a 33‐year‐old man. Threshold segmentation and Boolean operations were used to extract images of the ankle bones and build a three‐dimensional model (Mimics software, Materialise NV, Belgium). The bones were set as homogeneous and isotropic linearly elastic materials, with an elastic modulus of 17 GPa and a Poisson's ratio of 0.3; the elastic modulus of the articular cartilage was set to 0.4 GPa with a Poisson's ratio of 0.4; and the elastic modulus of the screws was set to 200 GPa with a Poisson's ratio of 0.3. Spring elements were used to simulate the ligaments with the following stiffness values: 600 N/mm for the interosseous membrane, 141.8 N/mm for the anterior talofibular ligament, 82 N/mm for the posterior talofibular ligament, 126.6 N/mm for the calcaneofibular ligament, and 7 N/mm for the suture anchor. Three‐dimensional models of the bones and screws were discretized into tetrahedral elements. The DL augmentation model and the screw placement model are shown in Figure 1. The frictional resistance between the articular surfaces of the bones was set as frictionless. The contact relationship between the screws and the fibula and tibia was set as bonded. The proximal ends of the tibia and fibula were completely fixed. During external rotation, a longitudinal pressure of 150 N was applied, and a torque of 4 N·m was applied at the center of the sole of the foot. Under the eversion working condition, a force of 60 N was applied to the medial side of the foot to make the foot evert under the action of the force.

### 2.3 Biomechanical Experiments

The biomechanical tests were performed on nine formalin‐fixed unpaired cadaveric lower limbs disarticulated at the knee joint (from the tibial plateau to the foot) from individuals with a mean age of 84.7 years (range, 81–87 years). The participants had no history of musculoskeletal injury or operation to the lower extremity. The specimens were prepared by first removing the soft tissues surrounding the ankle and lower leg while preserving the ligaments and the interosseous membrane. The forefoot was then removed, whereas the proximal tibiofibular joint was unconstrained to facilitate normal motion of the fibula relative to the tibia. The subtalar joint was solidly stabilized on each specimen using K‐wires to prevent additional subtalar joint movement. Before simulating the unstable ankle injury, the horizontal hole through which the trans‐syndesmotic screw was placed was pre‐drilled using a 2.5‐mm drill through the fibula and tibia to help reduce the syndesmosis anatomically later. An ankle injury model with both syndesmotic disruption and DL injury was constructed by sectioning the inferior tibiofibular ligament (including the anterior inferior tibiofibular ligament, posterior inferior tibiofibular ligament, and interosseous ligament), the tibiofibular interosseous membrane up to 6 cm proximal from the tibial plafond, and the superficial and deep DL. Because rigid plate and screw fixation is often used to treat lateral malleolus fractures, the fibula was left intact to simulate the rigid fixation applied after fibular fracture.

The external rotation and eversion stress tests were simulated under three conditions: intact condition before the DL and all syndesmosis ligamentous structures were cut, DL augmentation alone, and syndesmotic fixation alone. Each specimen was assessed using the two stress tests under all three conditions. The order of the two intervention measures and tests was randomized.

**FIGURE 2:**
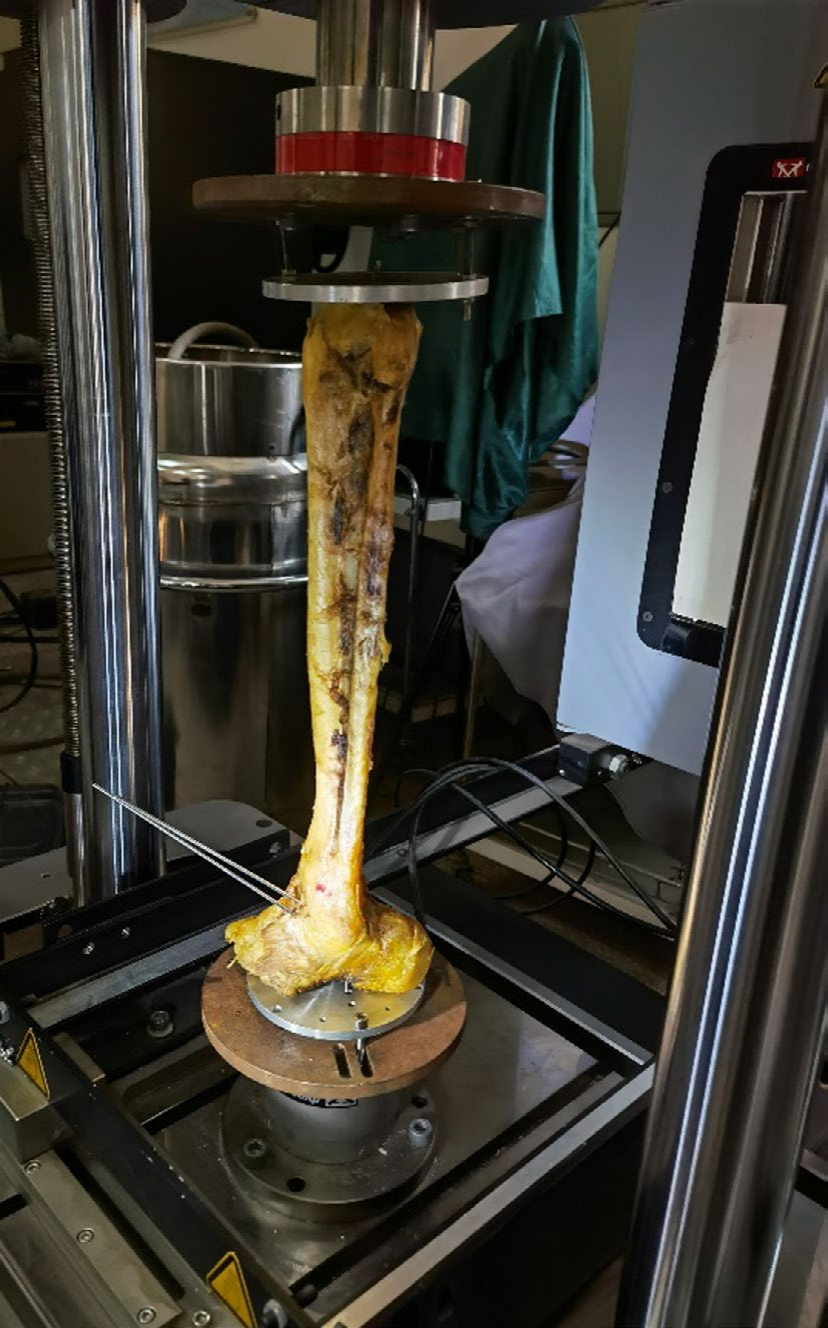
External rotation test. The tibial plateau was solidly fixed by K‐wires on the custom‐built clamps fastened to a hydraulic loading frame. The hindfoot was secured to custom‐built clamps in a neutral position using K‐wires. A compressive load of 150 N was applied along the long axis of the tibia in 2 min and was maintained throughout each trial. The tibia was internally rotated at a rate of 1 degree/s until a resistive moment of 4.0 N·m was reached.

In the external rotation stress test, the tibial plateau was solidly fixed by K‐wires on custom‐built clamps fastened to a hydraulic loading frame (ElectroPuls E10000; Instron, Norwood, MA, USA). The hindfoot was secured to custom‐built clamps in a neutral position using K‐wires (Figure 2). A compressive load of 150 N was applied along the long axis of the tibia in 2 min and was maintained throughout each trial. The tibia was internally rotated at a rate of 1 degree/s until a resistive moment of 4.0 N·m was reached, in accordance with a non‐destructive protocol [28, 29, 30, 31].

**FIGURE 3:**
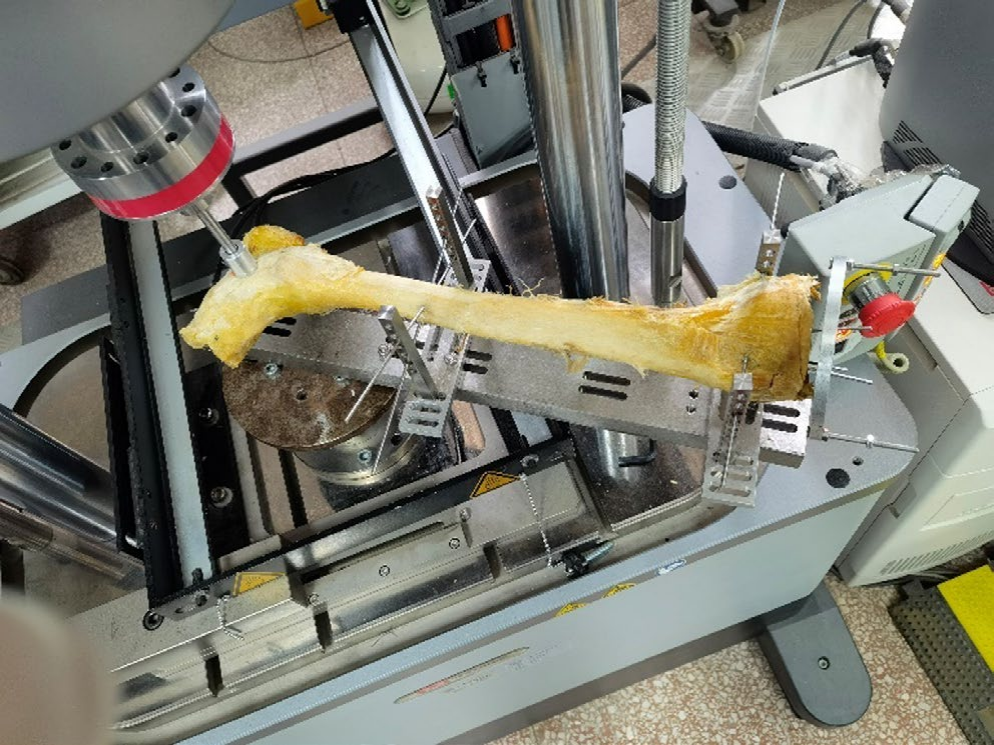
Eversion stress test. The tibia was fixed horizontally by K‐wires on the custom‐built frame. The plunger of the hydraulic loading frame was positioned 4 cm from the center of the ankle joint space. A vertical force was applied at a rate of 6.25 N/s until it reached 62.5 N, making the eversion torque 2.5 N·m.

In the eversion stress test, the tibia was fixed horizontally by K‐wires on the custom‐built frame (Figure 3). The plunger of the hydraulic loading frame was positioned 4 cm from the center of the ankle joint space. A vertical force was applied at a rate of 6.25 N/s until the force reached 62.5 N, making the eversion torque 2.5 N·m, as used in previous studies [28].

### 2.4 Outcome Measures

Vernier calipers were used to measure the following parameters at the beginning and end of each trial: medial clear space (MCS; distance between the talus and medial malleolus 5 mm below the superior margin of the talus) and tibiofibular clear space (TCS; distance between the tibia and the internal margin of the fibula 1 cm above the tibial articular surface). The MCS of the intact specimen was not measured because the superficial DL blocked the measurement. The external rotation angle of the joint was recorded, and the eversion angle was calculated using the descending distance of the pressure plunger. All measurements were carried out twice by the same researcher and averaged.

### 2.5 Statistical Methods

The data were described as medians and quartiles because they were not normally distributed. The parameters were compared using the Wilcoxon signed‐rank test for paired samples. *p* < 0.05 was taken to indicate statistical significance in all tests. All analyses were performed using R (R Core Team, 2023). The statistical analysis was conducted by the authors. The study was approved by the ethics committee of our hospital (approval number 201805–07).

## 3 Results

**FIGURE 4:**
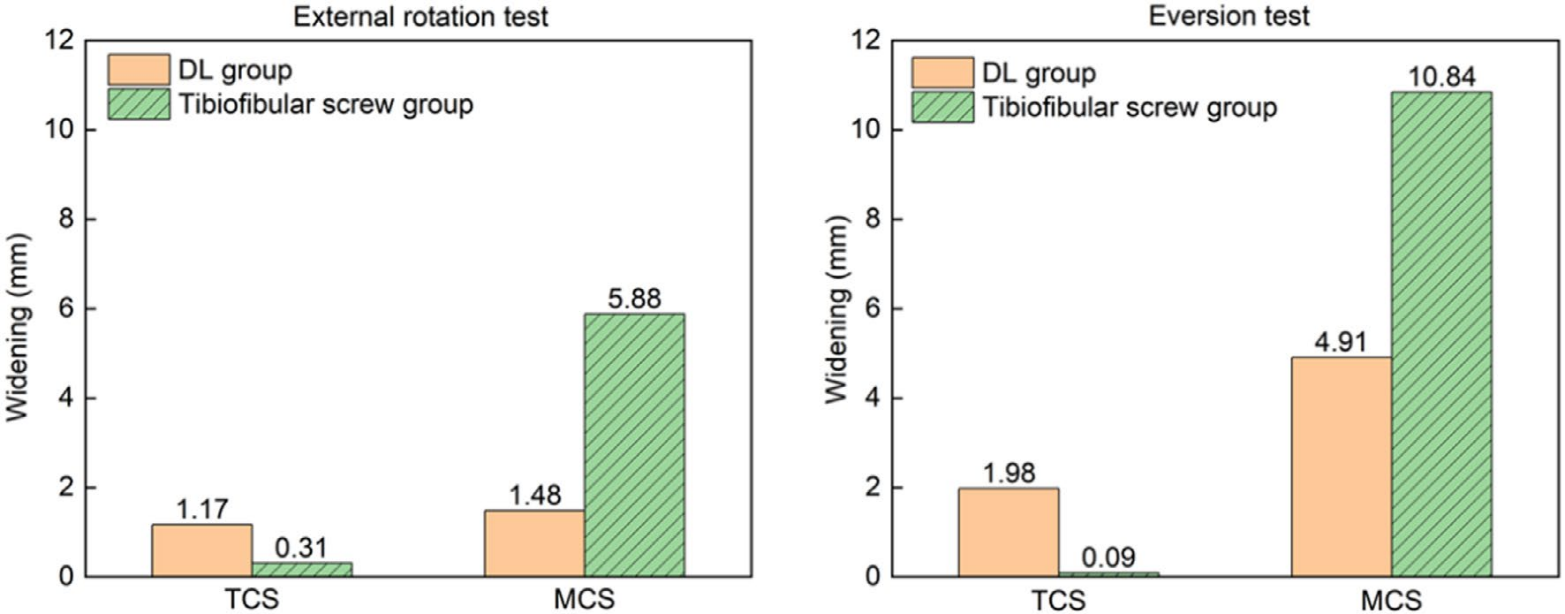
Comparison of the simulated displacement results during external rotation and eversion tests.

In the finite element study, the MCS widening was lesser and the TCS widening was greater in the DL augmentation group than in the syndesmosis fixation group in both the external rotation and eversion tests (Figure 4).

In the biomechanical tests, two of the nine specimens with syndesmotic screw fixation experienced severe dislocation during testing (one during external rotation and the other during eversion), causing the termination of the experiment. After both the external rotation and eversion tests, the MCSs and TCSs were significantly increased in both the DL augmentation and syndesmosis fixation groups (*p* < 0.05) (Table 1). There were no significant changes in the TCS in the intact specimens after rotation (*p* = 0.086, HLD = 0.37, 95% CI: −0.26~0.71) and eversion (*p* = 0.138, HLD = 0.13, 95% CI: −0.37~0.24) tests. The external rotation and eversion test results of the two groups are shown in Table 2. There were also no significant differences between the two groups in the widening of the TCS (*p* = 0.093, HLD = −0.79, 95% CI: −1.70~0.27) and in the rotation angle (*p* = 0.401, HLD = −1.99, 95% CI: −2.64~18.47) and eversion angle (*p* = 0.176, HLD = 2.85, 95% CI: −1.64~10.42). However, the widening of the MCS was significantly lesser in the DL augmentation group than in the syndesmotic fixation group during the rotation (*p* = 0.036, HLD = 3.57, 95% CI: 0.40~6.41) and eversion tests (*p* = 0.018, HLD = 4.36, 95% CI: 1.84~7.35).

**TABLE 1:** Comparison of measurements before and after external rotation and eversion tests.

	Before rotation/eversion	After rotation/eversion	HLD (95% CI)	Z value	*p*
External rotation tests					
Intact TCS (mm)	7.1 (6.3, 10.8)	7.5 (6.7, 10.8)	0.37 (−0.26~0.71)	1.718	0.086
DL MCS (mm)	9.8 (7.6, 10.8)	14.5 (12.1, 17.1)	5.32 (3.70~6.95)	2.666	0.008
DL TCS (mm)	8.3 (6.9, 12.3)	11.7 (8.4, 16.9)	2.78 (1.68~4.37)	2.666	0.008
Screw MCS (mm)	10.8 (7.7, 12.3)	19.8 (15.2, 23.3)	9.22 (6.48~11.97)	2.521	0.012
Screw TCS (mm)	7.0 (6.4, 9.9)	9.9 (7.0, 12.3)	1.86 (0.54~3.05)	2.521	0.012
Eversion tests					
Intact TCS (mm)	7.2 (6.6, 10.7)	7.5 (6.1, 10.9)	0.13 (−0.37~0.24)	1.483	0.138
DL MCS (mm)	9.2 (8.1, 11.3)	10.4 (9.0, 13.2)	1.34 (0.23~2.46)	2.100	0.036
DL TCS (mm)	8.0 (7.0, 12.4)	9.6 (7.0, 13.2)	1.38 (0.23~2.87)	2.240	0.025
Screw MCS (mm)	15.4 (13.8, 17.0)	21.3 (18.6, 25.2)	5.96 (2.43~8.77)	2.366	0.018
Screw TCS (mm)	7.3 (6.5, 11.2)	7.2 (6.8, 11.5)	0.39 (−0.09~1.42)	2.197	0.028

*Note*: Values are expressed as median and quartile. Significant *P* values are typed in bold. HLD (95% CI): Hodges–Lehmann difference (95% confidence interval).

**TABLE 2:** Comparison of measurements in external rotation and eversion tests.

	DL augmentation	Syndesmotic fixation	HLD (95% CI)	*Z* value	*p*
External rotation tests					
MCS widening (mm)	5.4 (3.7, 6.9)	9.4 (5.8, 10.7)	3.57 (0.40~6.41)	−2.100	**0.036**
TCS widening (mm)	2.8 (2.1, 4.6)	1.8 (0.5, 3.6)	−0.79 (−1.70~−0.27)	−1.680	0.093
Rotation angle (°)	41.8 (28.9, 54.7)	45.6 (30.2, 55.9)	1.99 (−2.64~18.47)	−0.840	0.401
Eversion tests					
MCS widening (mm)	1.3 (0.2, 2.5)	6.0 (3.5, 8.4)	4.36 (1.84~7.35)	−2.366	**0.018**
TCS widening (mm)	0.9 (0.1, 2.9)	0.3 (0.1, 1.0)	−0.84 (−2.57~1.09)	−1.183	0.237
Eversion angle (°)	17.4 (11.1, 23.2)	22.6 (17.5, 25.4)	2.85 (−1.64~10.42)	−1.352	0.176

*Note*: Values are expressed as median and quartile. Significant *p* values are typed in bold. HLD (95% CI): Hodges–Lehmann difference (95% confidence interval).

## 4 Discussion

Anatomical reconstruction of the joint is critical for obtaining favorable clinical results [32]. Recently the authors have been developing a novel approach to achieve mortise stability using deep DL augmentation alone that aimed to provide medial column stability, indirectly reduce the syndesmosis, and achieve primary stability of the ankle. The present study verified the effectiveness of this method from the perspective of biomechanics and found that compared with tibiofibular screw fixation, DL augmentation may better resist the widening of the MCS and achieve a similar effect in resisting the widening of the TCS.

### 4.1 Widening of the MCS

In both the external rotation and eversion tests of the present finite element experiment and the biomechanical experiment, the widening of the MCS was significantly lesser in the DL augmentation group than in the syndesmotic fixation group, suggesting that DL augmentation restored the stability of the tibiotalar joint around the medial malleolus better than syndesmotic fixation in both states. The reason for this phenomenon may be that DL augmentation constrained the talus directly, while tibiofibular screws indirectly constrained the talus. Additionally, DL augmentation directly restricted the external rotation and eversion movement of the talus, which mimicked the physiological effects of the DL. In the DL augmentation group, the MCS was better maintained, indicating that the talus was not dislocated outward and the tibiotalar relationship was well maintained during the experiment. This is the advantage of DL augmentation, as the important purpose of the surgery is to maintain the position of the talus and the tibiotalar relationship.

Although the need for surgical intervention for acute DL injury is still controversial [33], DL augmentation plays a significant role in ankle joint stability. DL is considered the primary stabilizer of the ankle, with the superficial and deep layers contributing mostly to eversion instability and external rotation instability, respectively [4]. A previous cadaver study showed that mild syndesmosis widening (less than 2 mm) occurs under external rotational stress with the medial malleolus and DL intact, although the syndesmosis and the interosseous membrane in this study were dissected 15 cm proximal to the ankle [34]. These previous results showed that when the DL was intact, the outward shift of the talus did not exceed 2 mm even if the distal fibula was absent, and there was no abnormal ankle movement [35]. In addition, previous biomechanical cadaveric studies have shown that isolated disruption of the anterior inferior tibiofibular ligament and interosseous ligament does not produce coronal instability, whereas concomitant DL disruption gives rise to instability [36, 37], suggesting the possible effect of the DL on the stability of tibiofibular syndesmosis. Deep DL augmentation is straightforward to perform, provides sufficient primary stability, and can be performed regardless of the avulsion level of the deep DL [3], so DL augmentation alone is a potential alternative to syndesmotic fixation regarding the stability of the medial malleolus.

### 4.2 Widening of the TCS

In the finite element experiment, the TCS widening tended to be lesser in the syndesmotic fixation group than in the DL augmentation group, and the *p*‐value of 0.093 for the TCS widening in the external rotation test was close to the significance threshold, which suggests that a significant difference may be revealed in a large sample size. This is because screw fixation can be regarded as rigid fixation, which better controls the TCS; however, we also believe that the TCS widening in the DL augmentation group was largely derived from the elasticity of the fibula, which allowed the talus to shift outward and permitted the slight separation of the tibiofibular syndesmosis. On the other hand, the lack of significant differences in the TCS widening between the two groups during the rotation and eversion biomechanical tests suggests that DL augmentation achieved some tibiofibular stability, similar to the syndesmotic fixation. This mechanism could be explained by the following two theories [1]. According to the three‐column theory, anatomical reduction and fixation of the fibula restore the overall stability of the ankle joint by repairing either the DL (medial column) or the tibiofibular syndesmosis (intermediate column) [2]. Syndesmotic fixation limits the transverse displacement of the talus by restricting the movement space without directly constraining the medial malleolus. Dislocation is prone to occur during eversion or external rotation of the ankle joint. However, the DL repair directly restricts the medial malleolus, preventing the outward movement of the talus and the resultant widening of the tibiofibular syndesmosis. Therefore, DL augmentation contributes to mortise stability in the presence of syndesmotic injury [5].

### 4.3 Rotation and Eversion Angles

In the biomechanical experiment, there were no significant differences between the two groups in the rotation and eversion angles. This phenomenon is inconsistent with the dislocation observed in some specimens without DL augmentation during external rotation and eversion, which could be due to the small sample size. However, although DL augmentation may directly constrain the talus, thereby avoiding excessive eversion and rotation of the talus, the tibiofibular screw may also limit such external rotation [28] by providing rigid fixation that eliminates the tibiofibular syndesmotic flexibility and reduces the rotation angle.

### 4.4 Innovations

Previous studies have investigated the stability of ankle fractures with ligamentous injury during DL augmentation and syndesmotic fixation, reporting similar clinical effects [23, 24, 25, 26], laying a foundation for the alternative treatment of syndesmotic fixation, but no study has elucidated the biomechanical mechanism. Previous studies have focused on degree IV Weber‐type B ankle fractures without medial malleolar fractures, wherein the fracture line is proximal to the tibiofibular syndesmosis, with the interosseous membrane exerting a stabilizing effect such that the tibiofibular stability is not destroyed completely. There are also biomechanical and imaging cadaveric studies [36, 37, 38]. Schottel et al. [30] found that posterior tibiofibular ligament repair with DL repair restored the ankle and syndesmotic rotational stability in a manner equivalent to that achieved with syndesmotic fixation. However, repairing the posterior tibiofibular ligament is a more complex approach that requires further soft tissue stripping, and this method still requires intervention in the tibiofibular syndesmosis. Presently, there is no biomechanical study on the simple use of DL augmentation alone instead of tibiofibular screw implantation for Weber‐ type C fractures.

The abovementioned studies have proven the critical role of the DL in ankle stability and provided some foundation for the present study. However, these previous studies did not evaluate the treatment of Danis‐Weber‐type C fractures for which there is a need to fix the tibiofibular syndesmosis (the “intermediate column”) [39] and do not include straightforward methods suitable for clinical application.

### 4.5 Clinical Relevance

This study is the first to find that it may be biomechanically possible to use DL augmentation alone instead of syndesmotic screw fixation for the treatment of ankle fractures with both DL rupture and syndesmotic diastasis. Compared with syndesmotic fixation, DL augmentation may better resist the widening of the medial malleolar space during eversion and external rotation and achieve an effect similar to tibiofibular screw placement in resisting the widening of the tibiofibular syndesmosis. Replacing the use of syndesmotic screw fixation in selected patients can reduce the need for fixation removal surgery, screw breakage, and screw misplacement. This study provides biomechanical evidence that indirect stabilization of the syndesmosis with DL augmentation may be a viable option for some Weber‐type C ankle fractures other than syndesmotic screws and tight‐ropes and provides novel biomechanic evidence of the important role of the DL in syndesmosis integrity.

### 4.6 Limitations

The present study has some limitations. Owing to the limited availability of specimens, we used formalin‐fixed rather than fresh‐frozen specimens. Formalin‐fixed samples have very rigid soft tissue, which may have affected the results. To combat this, we removed most of the soft tissue, leaving only the ligaments. As a previous study showed no significant differences between the experimental data of fresh and preserved specimens [34], the present study still holds great value. Another limitation is that although we found that the DL augmentation may have a comparable effect to syndesmotic fixation, this study didn't investigate the effect of repairing both DL and syndesmosis, which may restore more joint stability close to the native state [40].

In addition, the small sample size may have prevented some *p*‐values from reaching the level of statistical significance. Finally, the advanced ages of the cadavers and potential osteoporosis may have affected the accuracy of our results. Therefore, the present findings require validation in future biomechanical and clinical studies with larger sample sizes, and the cost‐effectiveness and surgical learning curve of this proposed method should be assessed.

## 5 Conclusion

Compared with syndesmotic fixation, DL augmentation has better resistance to medial malleolar space widening, under both external rotation and eversion forces, and can restore the tibiofibular space to a certain extent. These results suggest that DL augmentation alone is a potential alternative to syndesmotic fixation for Weber‐type C ankle fractures from a biomechanical point of view.

## Author Contributions

**Fei Han:** data curation, formal analysis, investigation, methodology, visualization, writing; **Liu Yan:** data curation, formal analysis, investigation, methodology; **Li Ting:** conceptualization, methodology, project administration, supervision; **Wang Jun:** data curation, formal analysis, investigation, methodology; **Sun Zhijian:** conceptualization, project administration; **Li Changrun:** investigation, methodology; **Zhang Weiguang:** resources, methodology; **Liu Huaicun:** resources, methodology; **Ding Huiru:** resources, methodology; **Huan Yong:** conceptualization, methodology, project administration, supervision, resources.

## Funding

This study was supported by the Capital's Funds for Health Improvement and Research (Code 2022‐2‐1121) and Beijing Hospitals Authority Clinical Medicine Development of Special Funding Support (Code: ZLRK202311).

## Ethics Statement

Ethical approval was obtained and checked from the Ethics Committee of our hospital (APPROVAL NUMBER 201805–07).

## Consent

The authors have nothing to report.

## Conflicts of Interest

The authors declare no conflicts of interest.

## Data Availability

The data that support the findings of this study are available from the corresponding author upon reasonable request.
